# Advertising Patrons

**Published:** 1871-04

**Authors:** 


### ADVERTISING PATRONS.

We take great pleasure in calling the attention of our readers to the advertisements of our business men. The Publisher has obtained for this department the patronage of some of the most reliable business houses in the city, and we trust that our readers may keep themselves posted in relation to these houses—with the character of their business—and when visiting the city, be certain to give the proprietors a call, with the assurance from us, that they will find them polite and accommodating gentlemen.

We must not omit, under this head, to allude, in the most favorable manner, to the establishment of Messrs. Sharpe & Dohme, Baltimore, Md. These gentlemen are largely engaged in manufacturing pure Chemicals for the use of the profession. Having tried their preparations, we have no hesitancy in pronouncing them reliable, and all they profess to be ; and would, especially, call the attention of Druggists to this firm, that in purchasing large supplies for our Southern markets, they may give these gentlemen a call. In our opinion they will obtain pure chemicals and on reasonable terms.

